# Draft Genome Sequence of *Rathayibacter* sp. Strain VKM Ac-2630 Isolated from Leaf Gall Induced by the Knapweed Nematode *Mesoanguina picridis* on *Acroptilon repens*

**DOI:** 10.1128/genomeA.00650-17

**Published:** 2017-07-27

**Authors:** Irina P. Starodumova, Sergey V. Tarlachkov, Natalia V. Prisyazhnaya, Lubov V. Dorofeeva, Elena V. Ariskina, Vladimir N. Chizhov, Sergei A. Subbotin, Lyudmila I. Evtushenko, Oleg V. Vasilenko

**Affiliations:** a All-Russian Collection of Microorganisms (VKM), G.K. Skryabin Institute of Biochemistry and Physiology of Microorganisms, Pushchino, Russia; b Pushchino State Institute of Natural Sciences, Pushchino, Russia; c Branch of Shemyakin and Ovchinnikov Institute of Bioorganic Chemistry, Pushchino, Russia; d Center of Parasitology, A.N. Severtsov Institute of Ecology and Evolution, Moscow, Russia; e California Department of Food and Agriculture, Sacramento, California, USA

## Abstract

A draft genome sequence of *Rathayibacter* sp. strain VKM Ac-2630 was derived using Ion Torrent sequencing technology. The genome size of this strain is 3.88 Mb, with an average G+C content of 72.0%. Genomic evidence of an aerobic mode of respiration and a heterotrophic lifestyle of this bacterium was obtained.

## GENOME ANNOUNCEMENT

The genus *Rathayibacter* (*Actinobacteria*) currently includes six validly described species, four of which (*R. rathayi*, *R. iranicus*, *R. tritici*, and *R. toxicus*) are plant pathogens transmitted to their host plants (wheat and grasses, *Poaceae*) by seed gall nematodes of the genus *Anguina* (*Anguinidae*) (1–3). The type strains of *Rathayibacter festucae* and *Rathayibacter caricis* have been isolated from *Festuca* *rubra* (*Poaceae*) infected by *Anguina graminis* and from *Carex* sp. (*Cyperaceae*) without any symptoms of nematode infestation (4). A few other new species have been identified but not validly described (5–7).

Strain VKM Ac-2630 was isolated from the leaf gall induced by the knapweed nematode *Mesoanguina* *picridis* (*Anguinidae*) on *Acroptilon* *repens* (*Asteraceae*) in Uzbekistan. The strain showed 99.79% 16S rRNA gene sequence similarity to the type strain of the phylogenetically closest species, *R. caricis* (7). However, the DNA-DNA hybridization value between these strains, obtained by a thermal denaturation method (8), was 56.8% ± 1.8%, which is below 70%, a threshold for circumscribing species genomically (9, 10). The strain also clustered separately from all recognized *Rathayibacter* species and from “*Rathayibacter tanaceti*” based on their matrix-assisted laser desorption ionization–time of flight (MALDI-TOF) mass spectra (7). The above data suggest that strain VKM Ac-2630 represents a novel species in the genus *Rathayibacter*.

The genome sequencing of VKM Ac-2630 was performed with the semiconductor genome analyzer Ion Torrent PGM (Thermo Fisher Scientific, Inc., USA) using a 400-bp sequencing kit and 318 v 2 chip. A total of 775,843 raw reads were assembled *de novo* into 136 contigs (34.0-fold peak coverage) using Newbler 2.9 (454 Life Sciences Corporation, USA). The genome size is 3,882,151 bp, with an average G+C content of 72.0%. The *N*_50_ contig was 56,149 bp, and the largest contig was 173,892 bp. The genome was annotated using NCBI PGAAP 4.0 (https://www.ncbi.nlm.nih.gov/genome/annotation_prok) and the RAST server 2.0 (10, 11). PGAAP identified 3,306 protein-encoding genes (1,613 of which encoded hypothetical proteins), 45 tRNAs, 3 rRNAs, and 3 noncoding RNAs (ncRNAs). The average gene length was approximately 900 bp, and the density was 0.985 genes per kb. Compared to PGAAP, the RAST server identified 3,744 protein-coding genes, 45 tRNAs, 3 rRNAs, and 370 function-related subsystems which cover 39% of predicted features.

The initial genome analysis of *Rathayibacter* sp. VKM Ac-2630 suggested that the organism is capable of utilizing carbohydrates, including l-arabinose, fructose, glucose, l-rhamnose, d-ribose, and xylose, as well as trehalose and glycogen. Genes for the biosynthesis of amino acids (methionine, glycine, proline, etc.), vitamins (thiamine, pyridoxin, etc.), and some compatible solutes (e.g., trehalose) were found. The enzymes characteristic of central metabolic pathways (i.e., glycolysis/glyconeogenesis, tricarboxylic acid cycle, pentose-phosphate pathway, and oxidative phosphorylation) were also detected, supporting an aerobic mode of respiration and a heterotrophic lifestyle of this bacterium.

Further genome-wide comparative analyses of VKM Ac-2630 and phylogenetically close bacteria, both pathogenic and nonpathogenic to plants, will facilitate insights into the nature and borderlines of prokaryotic species and also the understanding of molecular mechanisms involved in integrated effects of bacterial-nematode complexes on plants.

### Accession number(s).

This whole-genome shotgun project has been deposited at DDBJ/ENA/GenBank under the accession number MUKN00000000. The version described in this paper is the first version, MUKN01000000.
